# Preventing Smoking Relapse via Web-Based Computer-Tailored Feedback: A Randomized Controlled Trial

**DOI:** 10.2196/jmir.2057

**Published:** 2012-08-20

**Authors:** Iman Elfeddali, Catherine Bolman, Math J.J.M Candel, Reinout W Wiers, Hein de Vries

**Affiliations:** ^1^Department of Health PromotionSchool for Public Health and Primary Care (Caphri)Maastricht UniversityMaastrichtNetherlands; ^2^Department of PsychologyOpen University of the NetherlandsHeerlenNetherlands; ^3^Department of Methodology and StatisticsSchool for Public Health and Primary Care (Caphri)Maastricht UniversityMaastrichtNetherlands; ^4^Department of Developmental PsychologyUniversity of AmsterdamAmsterdamNetherlands

**Keywords:** Smoking relapse prevention, computer tailoring, multiple tailoring, planning strategies

## Abstract

**Background:**

Web-based computer-tailored approaches have the potential to be successful in supporting smoking cessation. However, the potential effects of such approaches for relapse prevention and the value of incorporating action planning strategies to effectively prevent smoking relapse have not been fully explored. The Stay Quit for You (SQ4U) study compared two Web-based computer-tailored smoking relapse prevention programs with different types of planning strategies versus a control group.

**Objectives:**

To assess the efficacy of two Web-based computer-tailored programs in preventing smoking relapse compared with a control group. The action planning (AP) program provided tailored feedback at baseline and invited respondents to do 6 preparatory and coping planning assignments (the first 3 assignments prior to quit date and the final 3 assignments after quit date). The action planning plus (AP+) program was an extended version of the AP program that also provided tailored feedback at 11 time points after the quit attempt. Respondents in the control group only filled out questionnaires. The study also assessed possible dose–response relationships between abstinence and adherence to the programs.

**Methods:**

The study was a randomized controlled trial with three conditions: the control group, the AP program, and the AP+ program. Respondents were daily smokers (N = 2031), aged 18 to 65 years, who were motivated and willing to quit smoking within 1 month. The primary outcome was self-reported continued abstinence 12 months after baseline. Logistic regression analyses were conducted using three samples: (1) all respondents as randomly assigned, (2) a modified sample that excluded respondents who did not make a quit attempt in conformance with the program protocol, and (3) a minimum dose sample that also excluded respondents who did not adhere to at least one of the intervention elements. Observed case analyses and conservative analyses were conducted.

**Results:**

In the observed case analysis of the randomized sample, abstinence rates were 22% (45/202) in the control group versus 33% (63/190) in the AP program and 31% (53/174) in the AP+ program. The AP program (odds ratio 1.95, *P *= .005) and the AP+ program (odds ratio 1.61, *P *= .049) were significantly more effective than the control condition. Abstinence rates and effects differed per sample. Finally, the results suggest a dose–response relationship between abstinence and the number of program elements completed by the respondents.

**Conclusion:**

Despite the differences in results caused by the variation in our analysis approaches, we can conclude that Web-based computer-tailored programs combined with planning strategy assignments and feedback after the quit attempt can be effective in preventing relapse 12 months after baseline. However, adherence to the intervention seems critical for effectiveness. Finally, our results also suggest that more research is needed to assess the optimum intervention dose.

**Trial Registration:**

Dutch Trial Register: NTR1892; http://www.trialregister.nl/trialreg/admin/rctview.asp?TC=1892 (Archived by WebCite at http://www.webcitation.org/693S6uuPM)

## Introduction

Smoking relapse rates can be extremely high (up to 90% in the first 3 months), and only 3%-5% of quitters maintain their quit attempt for 6 months or longer [1]. The role of risk factors for relapse (eg, low self-efficacy, the expectation of negative outcomes from quitting, negative affect, stress, and physical dependence) is quite well documented (see for examples [2-6]). However, a Cochrane review concluded that current smoking relapse prevention programs are not effective [7]. This ineffectiveness, combined with the alarming relapse rates, underlines the need for new, effective smoking relapse prevention strategies and programs. One potential explanation for these programs’ lack of effectiveness is that quitters are not fully prepared for their cessation attempt and lack sufficient coping strategies to maintain their attempts successfully [8-10].

Adding action planning components to programs may be a promising strategy for improving smoking relapse prevention programs [8-12]. Planning strategies are already incorporated into many face-to-face and telephone smoking cessation counseling sessions (eg, smoking cessation courses provided by the Dutch Foundation for a Smoke-free Future in the Netherlands) [13]. Furthermore, the role of action planning strategies is acknowledged by integrative social cognitive models, such as the Integrated Change (I-Change) Model [14,15]. The I-Change Model distinguishes between two essential planning components: (1) preparatory plans as actions designed to prepare for the behavior change, and (2) coping plans as actions designed to maintain the new behavior by coping with challenging or difficult situations. Yet, as far as we know, these planning strategies have received little emphasis in Web-based prevention programs.

The Internet has proven to be a promising delivery mode for health-promoting and lifestyle-changing interventions (for instance, [12,16-18]). Since 91% of Dutch households have access to the Internet, it could potentially be used to reach large numbers of people [19]. Additionally, interactive, personalized Internet-based delivery modes, such as computer tailoring, have already demonstrated their potential to support smoking cessation [12,20-23]. These highly personalized approaches are assessment based and adapt their messages to the needs of the respondent [24,25]. Compared with nontailored messages, tailored messages are evaluated more positively, attract more attention, and are more likely to be read [12,16,24,26]. Computer-tailored health programs have shown to be promising tools for promoting healthy behavior in general [27-32] and smoking cessation specifically [12,20-23], with multiple tailoring moments being more effective than a single tailoring moment [32-34]. Few computer-tailored programs have explicitly targeted smoking relapse (see Borland et al [35] for an example). In line with earlier studies [31,33,34], Borland’s study also indicated the surplus value of multiple tailoring moments and suggested a dose-response relationship between the number of feedback letters and smoking abstinence. Moreover, no studies have assessed the effects of using planning strategies in combination with multiple tailored feedback time points after the quit attempt.

In sum, the main goal of the Stay Quit for You (SQ4U) study was to assess the efficacy of two relapse prevention programs: (1) an action planning (AP) program that provided tailored feedback based on the baseline questionnaire and 6 preparatory and coping planning assignments, and (2) an action planning plus (AP+) program that extended the AP program by providing tailored feedback at 11 time points after the quit date. The efficacy of the programs was compared with that of a control group (with no intervention). Moreover, we aimed to assess possible dose–response relationships between abstinence and adherence to the number of program elements. First, we expected both programs to be more effective than the control condition in fostering continued abstinence 12 months after the start of the study (hypothesis 1). We expected the AP+ program to be the most effective. Moreover, we expected to find a dose–response relationship between continued abstinence and intervention dose (hypothesis 2). Finally, we will provide an overview of the respondents’ program evaluations.

## Methods

Ethics approval was obtained from the Medical Ethics Committee of Maastricht Academic Hospital and Maastricht University (MEC 08-3-003; NL21414.068.08). The study is registered with the Dutch Trial Register (NTR1892).

### Respondents and Recruitment

**Figure 1:**
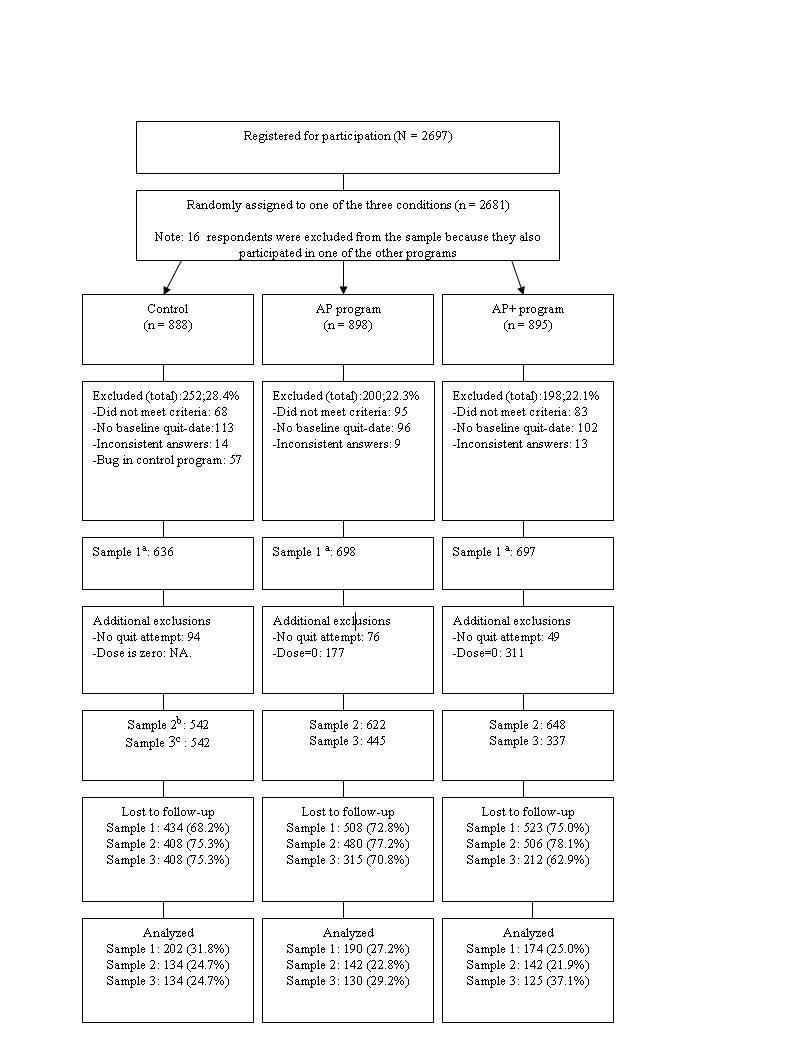
Enrollment and inclusion of respondents. NA = not applicable. ^a^ All respondents as randomly assigned, ^b^ modified sample excluding respondents who indicated after their quit date as well as at follow-up that they did not make a quit attempt during the study and respondents who quit too close to follow-up (see Analyses section in the Methods), ^c^ minimum dose sample additionally excluding respondents who did not adhere to at least one intervention element.

We recruited smokers by placing ads in local newspapers, distributing 10,000 flyers in the city of Maastricht, and placing online ads on the websites of national health funds, a national news page, and the Dutch Foundation for a Smoke-free Future. The ads referred the respondents to our research website for more information. All data were gathered via the Web and there was no face-to-face contact between respondents and the study team. A software program randomly assigned a total of 2681 respondents to one of the three conditions using a simple randomization type (see design below). The enrollment and inclusion of respondents is presented in Figure 1. Respondents were eligible for participation when they met the baseline inclusion criteria (aged 18–65 years, smoked daily, willing to set a quit date within 1 month, and motivated to quit smoking) and agreed with the informed consent. The final sample consisted of 2031 respondents, of whom 566 (27.98%) responded to the 12-month follow-up measurement.

### Design and Procedure

The SQ4U study was a randomized controlled study with a control condition and two experimental conditions. Respondents registered via the research website and made their own login account (each time they were invited for participation they received this account in the invitation mail). After registration, respondents were randomly assigned to one of the three programs outlined in the Introduction. Respondents in the intervention groups were invited by email or text message (optional) to perform intervention tasks (eg, filling out a planning assignment). The same procedure was used to invite all respondents for the 12-month follow-up measurement. Reminder emails were sent when respondents did not respond to the follow-up measurement, which asked them to report on their smoking behavior using self-assessed questionnaires. Respondents who reported that they were abstinent were asked to take biochemical validation tests (see below). Respondents who completed all parts of their assigned SQ4U variant (including those in the control group) were eligible to win 1 of 20 prizes of €250. A more detailed description of the development, design, study course, biochemical validation, and intervention elements can be found elsewhere [36].

### Biochemical Validation

We used cotinine assessments to validate reports of abstinence in a subsample (n = 70) of the respondents. This verified the nonsmoking status self-reported by respondents at the 12-month follow-up measurement. Only 1 (1%) respondent showed positive cotinine results and admitted to having smoked. Another respondent admitted to smoking when invited for biochemical validation. The smoking status for these 2 (3%) respondents was changed to relapse.

### Intervention Materials

#### Baseline Computer-Tailored Feedback Before the Quit Attempt (AP and AP+ programs)

Feedback focused on respondents’ perceptions of smoking and quitting and was based on previously tested effective computer tailoring programs that used the I-Change Model [12,20]. The feedback was intended to increase the respondents’ motivation to quit smoking and to prepare them for the period after the quit attempt. It targeted the pros and cons of not smoking and relapses, provided tips to enhance or increase self-efficacy, offered suggestions for dealing with social influences, explained the importance of preparatory planning, and gave ideas for preparing for the quit attempt and dealing with stress.

#### Planning Strategy Assignments Before and After the Quit Attempt (AP and AP+ programs)

Since planning assignments have proven to be promising strategies to foster cessation and prevent relapse, the SQ4U programs incorporated assignments designed to motivate respondents to use these strategies to make a quit attempt (first 3 assignments, prior to quit date) and to prevent relapse (final 3 assignments, after quit date). Two assignments focused on making and carrying out preparatory plans; 2 more assignments addressed coping planning. Finally, 1 assignment focused on a specific coping plan (making an emergency plan for coping in case of a lapse) and 1 assignment required making a specific preparatory plan (planning a reward for abstaining from smoking for some time).

#### Tailored Feedback After the Quit Attempt (AP+ program only)

Studies that use ecological momentary assessments to gain in-depth, day-to-day information on the process of quitting have noted that low self-efficacy and negative affect preceded lapsing [37-40]. Therefore, we targeted self-efficacy, recovery self-efficacy, and negative affect in the feedback moments after the quit attempt. We also included feedback about the level of planning. Each measurement after the quit attempt targeted two of the four concepts mentioned. Tailored feedback about each of the four concepts was provided daily during the first 3 days (after the quit date), once a week during the rest of the first month, and once every 2 weeks until the third month. The feedback not only addressed the respondents’ present state but was also ipsative, referring to earlier states (reporting changes in a positive or negative direction). Finally, all feedback targeted self-reported smoking behavior for the last three measurements and reported the progress of maintenance or decline over those three measurements.

### Baseline Questionnaire

The following basic information was collected about each respondent:

- *Demographic variables *assessed age, gender, and educational level (1 = low [no education or only primary or primary-level vocational education], 2 = medium [secondary or secondary-level vocational education], and 3 = high [higher education]).
- *Perceived stress *was assessed by 5 items based on the Perceived Stress Scale [41] and was rated on a 5-point scale (1 = never, 5 = very often). The mean scale score was included in the analyses (Cronbach alpha = .75).
- *Level of depression *was measured by 10 items (eg, “I feel anxious”) using the Center for Epidemiologic Studies Depression scale [42]. The answers were given on a 4-point scale (1 = never, 4 = all the time). The mean scale score was included in the analyses (alpha = .83).
- *Level of dependence *was assessed by 5 items (eg, the time when tobacco is smoked after awakening) based on an abbreviated Fagerström test [43,44]. The sum score was used in the analyses (0 = not dependent, 9 = very dependent).

The following questions about intention and motivational factors were based on questions used in previous studies [9,12,20,45-47]:

o *Attitudes *were measured on a 4-point scale (1 = no, 2 = yes, a bit, 3 = yes, 4 = yes, a lot/very) with four subscales measuring the following: (1) pros of not smoking, expressed by 9 statements representing positive expected outcomes (eg, “not smoking will save me money”) (alpha = .73), (2) cons of not smoking, expressed by 9 statements representing negative expected outcomes (eg, “not smoking will cause me more stress”) (alpha = .71), (3) pros of relapse, expressed by 4 statements representing positive expected outcomes (eg, “relapse will make me feel relieved”) (alpha = .82), and (4) cons of relapse, expressed by 6 statements representing negative expected outcomes (eg, “relapse will make me feel weak”) (alpha = .85).

o *Social support *was measured by 5 items that asked about the support received from significant others (partner, children, other family members, friends, or colleagues) on a 4-point scale (1 = no, 2 = yes, some, 3 = yes, moderate, and 4 = yes, a lot). A “not applicable” response was coded as missing. We created an index and used these scores in the analyses.

o *Modeling *was assessed by 5 items that measured the smoking status of the partner (yes, no, not applicable) and residential children, parents, colleagues, and friends (all, the majority, half, a minority, none, not applicable). The items were coded into three category scales (–1 = not smoking, 0 = not applicable, 1 = smoking). The items were summed and formed an index that we used in the analyses; smoking status of the partner was included separately in the analyses.

o *Self-efficacy *was assessed by 13 items that asked whether respondents thought they would be able to refrain from smoking in various smoking-related high-risk situations such as parties (alpha = .88). Recovery self-efficacy was assessed by 7 items that asked whether respondents thought they would be able to resume a smoking cessation program after a lapse (eg, after a week of abstinence) (alpha = .92). Answers were given on a 5-point scale (1 = totally disagree, 5 = totally agree) and the mean score was used in the analyses.

o *Preparatory planning *was assessed by 12 items (based on previous studies [8,9,12,46]) asking respondents to indicate whether they had plans to perform preparatory behaviors, such as removing all smoking materials, on a 5-point scale (1 = totally disagree, 5 = totally agree) (alpha = .72). The mean scale scores were used in the analyses.

#### Follow-up Measurements

Continued abstinence at 12 months after baseline was the primary outcome. In line with the definitions provided by Hughes et al [48], we defined continued abstinence as not smoking at all after the quit attempt. We measured it by asking whether the respondent had smoked since the quit date. Continued abstinence was coded as 0 = relapse and nonsmoking as 1 = abstinence.

### Analyses

The most common approach to analyzing the effects of interventions is to include all respondents randomly assigned to the programs. The advantage to this approach is that it reflects the situation as it is likely to occur in practice (where low adherence levels and not following program protocols will also be common). This strategy also maintains the balance between randomly assigned groups and preserves sample sizes [49,50]. On the other hand, including all randomly assigned respondents is a very conservative approach that may be susceptible to type II errors [49,50] and may say little about the efficacy of a treatment, since respondents who did not receive the treatment or did not follow the protocol are still included [49,51]. A modified approach (which has been used more frequently in the last decades [52]) seems to be more suitable for gaining insight into the effects of programs among those who actually followed them. However, excluding respondents after randomization may threaten the randomization balance and neglect the fact that nonadherence (and even making or not making a quit attempt) may also be a result of the treatment [50]. Therefore, both approaches have their advantages and disadvantages. To provide a complete overview of our results, we analyzed three samples and report on the above-mentioned modified approach. This resulted in three different analytic approaches with three different samples:

- Sample 1 is the *sample as randomized,* including all respondents. Since this study focused on relapse prevention and therefore on continued abstinence, we also report on smaller samples:
- Sample 2, referred to as *the modified sample,* excluded respondents who indicated after their quit date as well as at follow-up that they did not make a quit attempt, and those who reported that they had quit less than 320 days before the follow-up measurement (this is after about 2 weeks after the first month in which they were supposed to make an attempt). We excluded the latter group because one of the inclusion criteria was that respondents needed to set a quit date within 1 month after inclusion. Including respondents who explicitly stated that they made their quit attempt much later or even too close to the follow-up measurement would result in differences between respondents in the time frame from the quit attempt to the follow-up measurement.
- Sample 3, referred to as the *minimum dose sample,* excluded respondents who did not adhere to at least one element of the program they were assigned to (ie, did not complete the prequit and postquit assignments) in addition to meeting the exclusion criteria in the modified sample.

For all three samples, we conducted an observed case analysis (only including respondents with follow-up data) and a conservative analysis in which we assumed that participants missing at follow-up had relapsed to smoking. We used a logistic regression analysis to compare the efficacy of the control condition and the SQ4U programs to foster continued smoking abstinence after 12 months. Differences in baseline factors (demographics, smoking-related factors, perceived stress, depression, intention strength, social influence, attitude, self-efficacy, recovery self-efficacy, and preparatory planning) were assessed using chi-square tests for categorical variables and analysis of variance for continuous variables. Attrition was analyzed by logistic regression and included baseline factors. To preserve power, we included only baseline factors on which the three conditions significantly differed at baseline as well as the factors that significantly predicted dropout at follow-up as covariates in the effect analyses. Listwise deletion with regard to missing values was used. Finally, chi-square tests were used to assess the existence of dose-response relationships within the SQ4U programs using the observed cases in sample 2 (the modified sample) to conform to the methods used by Borland et al [35].

## Results

### Sample Characteristics and Attrition Check

Table 1 presents the demographic and smoking-related characteristics at baseline and shows no significant differences between these variables in the three conditions. The mean age of the respondents was 41 (SD 11.80) years, and 1265 of the 2031 (62.28%) respondents were female. On average, respondents smoked 20 (SD 8.73) cigarettes per day, and 1887 (92.91%) had made previous attempts to quit. Furthermore, we found no significant (*P *> .05) differences between the three conditions on the baseline measurements of perceived stress, depression, intention strength, social influence, attitude, self-efficacy and recovery self-efficacy, and preparatory planning. (Table 1 shows only demographics and smoking-related factors.)

 In the control condition, 434 of 636 (68.2%) were lost to follow-up versus 508 of 698 (72.8%) in the AP program and 523 of 697 (75.0%) in the AP+ program (Figure 1). Attrition analysis proved that dropout was more likely among respondents in the AP (odds ratio [OR] 1.26, *P *= .06) and AP+ (OR 1.44, *P *= .004) programs and respondents who were younger (OR 0.98, *P *< .001, 95% confidence interval [CI] 0.97–0.99), were male (OR 1.32, *P *= .02, 95% CI 1.05–1.66), had a lower education level (OR 2.00, *P *= .001, 95% CI 1.33–3.00), perceived fewer advantages to relapse (OR 0.80, *P *= .02, 95% CI 0.67–0.97), and had low levels of self-efficacy (OR 0.78, *P *= .001, 95% CI 0.67–0.91).

**Table 1:** Means and baseline differences between the three programs in demographic and smoking-related variables.

Characteristic		Overall (N = 2031)	Control (n = 636)	AP^a^ (n = 698)	AP+^b^ (n = 697)	*F *value/χ^2^	*P *value
Female gender, n (%)		1265 (62.3%)	381 (59.9%)	442 (63.3%)	442 (63.4%)	2.2	.33
Age (years), mean (SD)		40.88 (11.80)	40.68 (11.81)	40.75 (11.48)	41.18 (12.12)	0.36	.70
**Educational level, n (%)**						6.4	.17
	Low	207 (10.2%)	57 (9%)	86 (12%)	64 (9%)		
	Medium	1130 (55.6%)	357 (56.1%)	371 (53.2%)	402 (57.7%)		
	High	694 (34.2%)	222 (34.9%)	241 (34.5%)	231 (33.1%)		
Cigarettes smoked per day, mean (SD)		19.85 (8.73)	19.85 (8.39)	19.89 (9.36)	19.80 (8.41)	0.02	.98
Smoking duration (years), mean (SD)		24.81 (11.96)	24.61 (11.90)	24.79 (11.58)	25.01 (12.41)	0.19	.83
Previous quit attempts (yes), n (%)		1887 (92.9%)	588 (92.5%)	654 (93.7%)	645 (92.5%)	1.0	.61
Nicotine dependence, mean (SD)^d^		4.53 (2.18)	4.57 (2.21)	4.48 (2.13)	4.55 (2.19)	0.26	.77

^a ^Action planning.

^b ^Action planning plus.

^c ^Degrees of freedom (DF) = 2 for all except for educational level (DF = 4).

^d ^Sum score of abbreviated Fagerström test (0 = not dependent, 9 = very dependent).

### Abstinence Rates 12 Months After Baseline

Table 2 presents the abstinence rates for samples 1, 2, and 3 when using observed cases alone, as well as when conducting conservative analyses and considering dropouts as relapsers. When using the observed case analysis strategy on sample 1, abstinence rates in the control group, the AP program, and the AP+ program were 22%, 33%, and 31%, respectively (see Table 2). In the sample where those who did not make a quit attempt conforming to the study protocol were excluded (sample 2), we found abstinence rates of 34%, 44%, and 37%, respectively. Finally, abstinence rates among only those who adhered to at least one of the intervention elements from the program they were assigned to (sample 3) showed abstinence rates of 34%, 46%, and 39%, respectively.

**Table 2:** 12-month abstinence rates per program for the three samples using observed and conservative analyses.

Sample	Observed				Conservative			
	n	n (%) abstinent			n	n (%) abstinent		
	Total	Control	AP^a^	AP+^b^	Total	Control	AP	AP+
1^c^	566	45 (22%)	63 (33%)	53 (31%)	2031	45 (7%)	63 (9%)	53 (8%)
2^d^	418	45 (34%)	63 (44%)	53 (37%)	1812	45 (8%)	63 (10%)	53 (8%)
3^e^	389	45 (34%)	60 (46%)	49 (39%)	1324	45 (8%)	60 (14%)	49 (15%)

^a ^Action planning.

^b ^Action planning plus.

^c ^Including all respondents as randomly assigned.

^d ^Modified sample excluding respondents who indicated after their quit date as well as at follow-up that they did not make a quit attempt during the study and respondents who quit too close to follow-up (see Analyses section in the Methods).

^e ^Minimum dose sample additionally excluding those who adhered to none of the intervention elements of their SQ4U variant.

### Main Effects of the AP and AP+ Programs With Correction for Covariates

Table 3 presents the main effects of the AP and AP+ programs compared with the control condition, while controlling for covariates (factors that predicted dropout according to the attrition analysis). The table shows these findings for observed case analyses as well as conservative analyses for sample 1 (including all respondents as randomly assigned), sample 2 (modified sample), and sample 3 (minimum dose sample). The AP program was significantly more effective in fostering abstinence than was the control condition in sample 1 (OR 1.95, *P *= .005), sample 2 (OR 1.71, *P *= .04), and sample 3 (OR 1.84, *P *= .02) when including observed cases only. The AP+ program was, however, only significant in sample 1 (OR 1.61, *P *=.049). In the conservative analyses, both programs were significantly more effective than the control condition only in sample 3 (AP: OR 1.72, *P *= .01; AP+: OR 1.76, *P *= .01). Finally, having high levels of self-efficacy was the only consistent predictor of continued abstinence in all these analyses.

**Table 3:** Regression 12-month continued abstinence on the AP^a ^and AP+^b ^program in sample 1 (observed cases, n = 559; conservative analysis, n = 1974), sample 2 (observed cases, n = 412; conservative analysis, n = 1757), and sample 3 (observed cases, n = 383; conservative analysis, n = 1297).

Variable		Observed case analysis^c^			Conservative analysis^d^		
		OR^e^	95% CI^f^	*P* value	OR	95% CI	*P* value
**Sample 1** ^g^							
	Gender	1.15	0.77–1.71	.50	.91	0.64–1.29	.59
	Age	1.01	0.99–1.03	.26	1.02	1.01–1.04	.002
	Low education level (high^h^)	1.61	0.75–3.43	.22	.76	0.40–1.43	.40
	Pros of relapse	0.87	0.63–1.20	.39	1.00	0.76–1.32	.98
	Self-efficacy	1.51	1.14–1.99	.004	1.64	1.29–2.07	<.001
	AP program (control^h^)	1.95	1.23–3.11	.005	1.38	0.92–2.08	.12
	AP+ program (control^h^)	1.61	1.00–2.60	.049	1.12	0.73–1.70	.61
**Sample 2** ^i^							
	Gender	1.12	0.73–1.73	.59	.88	0.62–1.25	.48
	Age	1.01	0.99–1.03	.28	1.03	1.01–1.04	.001
	Low education level (high^h^)	1.69	0.73–3.95	.22	.71	0.37–1.34	.29
	Pros of relapse	0.81	0.57–1.15	.23	1.00	0.76–1.32	.99
	Self-efficacy	1.39	1.03–1.89	.03	1.62	1.28–2.06	<.001
	AP program (control^h^)	1.71	1.03–2.83	.04	1.29	0.86–1.95	.23
	AP+ program (control^h^)	1.22	0.73–2.03	.44	.99	0.65–1.52	.98
**Sample 3** ^j^							
	Gender	1.17	0.75–1.83	.49	.87	0.61–1.26	.47
	Age	1.01	0.99–1.03	.31	1.02	1.01–1.04	.005
	Low education level (high^h^)	1.95	0.81–4.72	.14	.87	0.45–1.68	.68
	Pros of relapse	0.86	0.60–1.23	.41	1.06	0.80–1.42	.68
	Self-efficacy	1.43	1.04–1.96	.03	1.53	1.19–1.97	.001
	AP program (control^h^)	1.84	1.10–3.07	.02	1.72	1.13–2.61	.01
	AP+ program (control^h^)	1.36	0.80–2.29	.26	1.76	1.13-2.73	.01

^a ^Action planning.

^b ^Action planning plus.

^c ^Sample including only respondents with follow-up data.

^d ^Sample including missing data at follow-up as treatment failures.

^e ^Odds ratio.

^f ^Confidence interval.

^g ^Including all respondents as randomly assigned.

^h ^Reference category.

^i ^Modified sample excluding respondents who indicated after their quit date as well as at follow-up that they did not make a quit attempt during the study and respondents who quit too close to follow-up (see Analyses section in the Methods).

^j ^Minimum dose sample excluding those who did not adhere to at least one of the SQ4U elements.

### Abstinence Rates per Program Stratified to Intervention Dose

Table 4 shows the 12-month continued abstinence rates per intervention dose for the modified sample (sample 2). As posed in hypothesis 2, the results revealed significant relationships between abstinence and the number of planning assignments using linear by linear association chi-square tests (AP: χ^2^_1 _= 7.4, *P *< .007; AP+: χ^2^_1 _= 14.7, *P *< .001) and feedback moments (AP+: χ^2^_1 _= 24.5, *P *< .001) and confirmed higher abstinence rates when more planning assignments or questionnaires after the quit attempt were completed. On average, respondents in the program groups adhered to 4 out of 6 planning assignments and 6 out of 11 feedback questionnaires.

**Table 4:** 12-month continued abstinence rates stratified by the number of planning assignments and feedback moments in the modified sample (sample 2).

Stratification	Dose	AP^a^		AP+^b^	
		n^c^	n (%) abstinent	n^c^	n (%) abstinent
Per number of assignments	0–1	27	6 (22%)	23	4 (17%)
	2–4	53	24 (45%)	49	13 (27%)
	5–6	62	33 (53%)	70	36 (51%)
	Total, mean (SD)		3.71 (2.00)		3.95 (1.93)
	χ^2^_1_		7.4		14.7
	*P *value		.007		<.001
Per number of feedback moments	0–5	NA^d^	NA	66	10 (15%)
	6–7	NA	NA	12	4 (33%)
	8–9	NA	NA	31	19 (61%)
	10–11	NA	NA	33	20 (61%)
	Total, mean (SD)		NA		5.77 (3.83)
	χ^2^_1_		NA		24.5
	*P *value		NA		<.001

^a ^Action planning.

^b ^Action planning plus.

^c ^Only complete cases.

^d ^Not applicable, as the AP program did not provide tailored feedback after the quit date.

### Program Evaluation of the AP and AP+ Programs

Table 5 presents the program evaluation that was conducted 6 months after baseline. Respondents from the AP and AP+ programs gave the baseline-tailored feedback a positive evaluation. However, significantly more respondents in the AP+ program remembered the content of the baseline-tailored feedback and perceived the feedback as relevant and helpful for making the quit attempt. The planning assignments were perceived as useful by 87 of 164 (53%) respondents. Moreover, 724 (44%) of the respondents rated them as helpful for making a quit attempt and 51 (31%) agreed they were helpful in maintaining a quit attempt. Respondents from the AP and AP+ programs did not differ significantly on the evaluation of the planning assignments. The feedback after the quit attempt (short feedback moments) was rated as useful by 66 of 104 (63%) respondents, as helpful for making a quit attempt by 49 (47%), and as helpful for maintaining the quit attempt by 50 (48%).

**Table 5:** Program evaluation (conducted 6 months after baseline) by respondents from the AP^a ^and the AP+^b ^programs.

Evaluation item			Total (n = 248)	AP (n = 137)	AP+ (n = 111)	χ^2^_2_	*P *value
**Baseline feedback**							
	Remembered the content					9.7	.008
		Yes	142 (57.3%)	72 (53%)	70 (63%)		
		Neutral	63 (25%)	32 (23%)	31 (28%)		
		No	43 (17%)	33 (24%)	10 (9%)		
	Perceived feedback as useful					4.4	.111
		Yes	156 (62.9%)	79 (58%)	77 (69%)		
		Neutral	68 (27%)	41 (30%)	27 (24%)		
		No	24 (10%)	17 (12%)	7 (6%)		
	Perceived feedback as relevant					8.8	.012
		Yes	121 (48.8%)	56 (41%)	65 (59%)		
		Neutral	98 (40%)	60 (44%)	38 (34%)		
		No	29 (12%)	21 (15%)	8 (7%)		
	Perceived feedback as understandable					3.8	.152
		Yes	208 (83.9%)	110 (80.3%)	98 (88%)		
		Neutral	34 (14%)	24 (18%)	10 (9%)		
		No	6 (2%)	3 (2%)	3 (3%)		
	Recognized own situation in feedback					0.2	.92
		Yes	117 (47.2%)	63 (46%)	54 (49%)		
		Neutral	101 (40.7%)	57 (42%)	44 (40%)		
		No	30 (12%)	17 (12%)	13 (12%)		
	Perceived feedback as credible					3.6	.16
		Yes	170 (68.5%)	87 (64%)	83 (75%)		
		Neutral	67 (27%)	43 (31%)	24 (22%)		
		No	11 (4%)	7 (5%)	4 (4%)		
	Feedback helped to make a quit attempt					6.7	.04
		Yes	107 (43.1%)	51 (37%)	56 (50%)		
		Neutral	73 (29%)	49 (36%)	24 (22%)		
		No	68 (27%)	37 (27%)	31 (28%)		
	Feedback helped to maintain quit attempt					3.7	.16
		Yes	72 (29%)	33 (24%)	39 (35%)		
		Neutral	65 (26%)	38 (28%)	27 (24%)		
		No	111 (44.8%)	66 (48%)	45 (41%)		
**Planning assignments**			n = 164	n = 93	n = 71		
	Perceived feedback as useful					4.0	.14
		Yes	87 (53%)	49 (53%)	38 (54%)		
		Neutral	62 (38%)	32 (34%)	30 (42%)		
		No	15 (9%)	12 (13%)	3 (4%)		
	Feedback helped to make a quit attempt					5.6	.06
		Yes	72 (44%	37 (40%)	35 (49%)		
		Neutral	57 (35%)	30 (32%)	27 (38%)		
		No	35 (21%)	26 (28%)	9 (13%)		
	Feedback helped to maintain quit attempt					5.1	.08
		Yes	51 (31%)	27 (29%)	24 (34%)		
		Neutral	56 (34%	27 (29%)	29 (41%)		
		No	57 (35%)	39 (42%)	18 (25%)		
**Feedback moments after quit attempt (n = 104)**							
	Perceived feedback as useful						
		Yes	66 (63%)	NA^c^	66 (63%)		
		Neutral	26 (25%)	NA	26 (25%)		
		No	12 (12%)	NA	12 (12%)		
	Feedback helped to make a quit attempt						
		Yes	49 (47%)	NA	49 (47%)		
		Neutral	32 (31%)	NA	32 (31%)		
		No	23 (22%)	NA	23 (22%)		
	Feedback helped to maintain quit attempt						
		Yes	50 (48%)	NA	50 (48%)		
		No	54 (52%)	NA	54 (52%)		

^a ^Action planning.

^b ^Action planning plus.

^c ^Not applicable.

## Discussion

The main goal of this study was to evaluate the efficacy of two Web-based smoking relapse prevention programs (AP and AP+) in the SQ4U study with regard to 12-month continued smoking abstinence. Furthermore, we aimed to assess dose-response associations. Despite the potential of the Internet, a recurrent problem in Internet trials is low adherence to the programs [53]. These low levels of adherence may lead to underestimations of intervention effects. This impression is strengthened by the fact that most studies of Internet interventions have shown dose-response relationships (see, for instance, [35,54-58]). Hence, an alternative strategy in which nonadherent respondents are excluded may be needed. Recently, a systematic review has discussed an increase in the literature of randomized controlled trials that report on a modified strategy [52]. To provide a complete overview of our results, we employed both approaches and reported on three samples: (1) the sample as randomly assigned, including all respondents, (2) a sample excluding those who did not make a quit attempt (modified sample), and (3) a sample additionally excluding those who did not adhere to at least one of the intervention elements (minimum dose sample). Observed case analyses (only including respondents with follow-up data) and conservative analyses (including all cases and coding respondents who were missing at follow-up as smoking relapsers) were conducted on all samples.

The results of all three observed case analyses (samples 1, 2, and 3) revealed significant effects in favor of the AP program. We can conclude that most of the analyses support our first hypothesis that planning strategies can be effective in preventing relapse among smokers who are motivated to quit smoking. The AP+ program, on the other hand, only proved to be significantly more effective than the control condition in the randomized sample (sample 1). The approach for sample 2 seems to have created a bias against the AP+ program by excluding respondents who did not make a quit attempt during the study, which resulted in more exclusions of relapsers (since nonquitters cannot be considered to be continued abstinent at follow-up) in the control condition than in the AP+ program (which consisted of elements designed to foster making a quit attempt). This approach may therefore have been too conservative. Furthermore, additional power analyses showed that 2623 respondents would have been needed to find significant differences in abstinence rate between the control group (45/134, 34%) and the AP+ program group (53/142, 37%). Therefore, the lack of significance in sample 2 may be due to power issues. Another possible explanation for lack of significant effect for the AP+ program in sample 2 and sample 3 may be that the program was too intensive and therefore resulted in an overload for the respondents that negatively influenced its efficacy. A comparable result was found in an earlier study conducted among vocational students, which tested the efficacy of a standard in-school program, a computer-tailored program, and a combined program [59]. This study found that the combined strategy did not have a surplus value. The authors suggested that combining strategies may lead to an overload of information, which may produce more negative effects. Although this may also account for our findings, program evaluation data do not support this suggestion because the AP+ and AP programs both received a positive evaluation. A final explanation may be that the planning assignments and the feedback provided after the quit attempt may not have been an ideal combination to foster abstinence after all.

When conducting conservative analyses, we found the AP and AP+ programs to be significantly more effective than the control condition in the minimum dose sample (sample 3). However, given the large dropout rate, this conservative approach (coding missing participants as relapsers) may be too conservative. Additional descriptive analyses indicated that about 60% of the respondents who dropped out at follow-up were nonsmokers at their last visit, indicating that interpreting all missing cases as treatment failures may be too conservative, a finding also supported by others [60]. Finally, conservative analyses in the minimum dose sample (sample 3) may have biased the results in favor of the experimental conditions by excluding nonadherers (whose follow-up data are generally missing). Excluding these nonadherers results in fewer imputations of relapsers in the experimental conditions than in the control condition, as these respondents are already excluded because they did not adhere to the programs.

The abstinence rate of 22% in the control group is another notable finding, since self-quitters generally reach abstinence rates of only 3%-5% [1]. A possible explanation for this high abstinence rate may be that respondents in our control condition, who were highly motivated to quit smoking, sought additional help themselves when they did not receive the help they expected from our program. Since not all respondents reported on their use of additional help, we were not able to test this assumption with the available data. Moreover, the high motivation of these respondents may have played a role in their high abstinence rates.

Finally, the dose-response relationship between abstinence and the number of program elements suggests that a dose-response association may exist. This is because abstinence rates increased by up to 53% (33/62) in the AP program and 51% (36/70) in the AP+ program when doing 5 to 6 planning assignments, and up to 61% (20/33) in the AP+ program when filling out 10-11 feedback questionnaires (on which feedback was provided). These findings suggest, in line with hypothesis 2, that the efficacy of the programs depends on adherence to the program, also found in previous studies [35,61]. Our findings regarding dose-response relationships may, however, be attributed to the fact that respondents who relapsed after a few sessions discontinued the program, resulting in the finding that those who continued with the program were more successful. Caution is, therefore, needed when interpreting dose-response relationships: these relationships can be subject to selection biases, since the respondents are not randomly assigned to different doses [62]. Additional research that tests the assumption of a dose-response relationship in this context is needed in the form of an experimental design in which respondents are randomly assigned to groups with different doses.

The SQ4U study was subject to limitations. The first limitation is that the planning assignments and feedback moments were provided at fixed times, while the varying levels of adherence found in our study suggest that programs should perhaps provide support when the respondent needs it most (ie, by real-time support in difficult situations). Research is needed to explore the potential additional efficacy of such an approach. Second, the cut-off point for the minimum dose sample (sample 3) is not based on empirical findings and needs to be explored in additional studies. Third, because of medical ethics guidelines, we could not prevent respondents from using additional help to quit smoking. The use of additional help, however, may interfere with the effects of the programs and may be beneficial or counterproductive. Further research is needed to explore which additional aids may have positive or negative effects. Fourth, our study had a high rate of loss to follow-up (1465/2031, 72.1%), an issue that is very common in comparable studies [12,21,63,64]. Attrition may have been caused by factors such as spam filters or invalid email accounts or because people who have quit smoking do not want to be reminded of their past smoking behavior [65]. The latter is partly supported by our data, which showed that about 60% of the respondents who dropped out of the experimental programs were nonsmokers at their last visit. Finally, the conservative analyses (in which missing answers at follow-up were regarded as relapse) may be too conservative, as our data showed that about 60% of the respondents who dropped out of the experimental programs were nonsmokers at their last visit. Furthermore, attrition analyses indicated that dropout was more likely in the experimental programs. Consequently, the conservative analyses can be subject to biases strengthening the relation between the experimental programs and relapse, while the actual relation is one between the programs and dropout. Therefore, caution is needed with interpreting these results.

Aside from these limitations, our SQ4U study is the first to test the efficacy of incorporating planning assignments in a Web-based computer-tailored relapse prevention program and of combining planning assignments with multiple tailored feedback moments after the quit date. The study reflected on the findings from different samples, and the results pointed out the importance of using planning strategies and tailored feedback moments after the quit date for smokers who are motivated to quit. Previous studies indicated that a lack of preparatory planning is associated with smoking relapse [8,9]. Our current study further illuminates the role of planning by showing that managing behavior using action planning strategies in relapse prevention programs fosters abstinence. Additional research is needed to determine the optimum dose for reaching best effects and which planning strategies are most effective for which groups. Since some of the respondents in the experimental programs did not adhere to even one intervention element, insight into the predictors of adherence and into strategies to facilitate adherence are also needed.

## Abbreviations

AP: Action planning (program)
AP+: Action planning plus (program)
CI: confidence interval
I-Change Model: Integrated Change Model
OR: odds ratio
SQ4U: Stay Quit for You

## Multimedia Appendix 1

CONSORT eHealth V1.6.1 checklist [66].
