# Supplementary material for: Preventing Smoking Relapse via Web-Based Computer-Tailored Feedback: A Randomized Controlled Trial
Source: J Med Internet Res. 2012 Aug 20;14(4):e109. doi: 10.2196/jmir.2057 (PMC3510689; doi:10.2196/jmir.2057)
Supplement: Supplementary file 1 [file jmir_v14i4e109_app1.pdf]

CONSORT-EHEALTH Checklist V1.6 Report	Manuscript Number	2057
Date completed	8/25/2011 14:50:29	
by	Iman Elfeddali	

"Preventing smoking relapse via web-based computer-tailored feedback in the SQ4U-study: a randomized controlled trial".

TITLE

1a-i) Identify the mode of delivery in the title

The title of the manuscript contains the term "web-based" to refer to the delivery mode of the programs used.

1a-ii) Non-web-based components or important co-interventions in title

1a-iii) Primary condition or target group in the title

Our study does not focus on a special population, but on adult daily smokers in general. Therefore, we did not explicitly mention the target population in the title of the manuscript.

ABSTRACT

1b-i) Key features/functionalities/components of the intervention and comparator in the METHODS section of the ABSTRACT

The features and components of the intervention are explained in the section 'objectives' of the Methods (abstract). Relevant sections: "The actionplanning (AP) program provided tailored feedback at baseline and invited respondents to make preparatory and coping plans six times during the program. The action planning plus (AP+) program was an extended version of the AP program that also provided tailored feedback at eleven time points after the quit-attempt. Respondents in the control group only filled out questionnaires."

1b-ii) Level of human involvement in the METHODS section of the ABSTRACT

1b-iii) Open vs. closed, web-based (self-assessment) vs. face-to-face assessments in the METHODS section of the ABSTRACT

1b-iv) RESULTS section in abstract must contain use data

1b-v) CONCLUSIONS/DISCUSSION in abstract for negative trials

INTRODUCTION

2a-i) Problem and the type of system/solution

In the Introduction it is explained that smoking relapse is a problem. Relevant sections: "Smoking relapse rates can be extremely high (up to 90% in the first three months) and only 3–5% of quitters maintain their quit-attempt for six months or longer" and "a Cochrane review concluded that current smoking relapse prevention programs are not effective." Furthermore, it is explained that we plan to target smoking relapse using a computer tailoring program incorporating planning strategies and that the program is supposed to be more successful in preventing smoking relapse.

2a-ii) Scientific background, rationale: What is known about the (type of) system

In the Introduction it is explained why these interventions are developed. Relevant sections: "Smoking relapse rates can be extremely high" and "current smoking relapse prevention programs are not effective". Furthermore, the sub parts (internet, planning components and computer tailoring) and the use of these components with regard to other behaviors and smoking cessation are described in the Introduction.

METHODS

3a) CONSORT

The final paragraph of the Introduction contains goals and hypotheses: "In sum, the main goal of the SQ4U-study is to assess the efficacy of two relapse prevention programs: (1) an action planning (AP) program that provides tailored feedback based on the baseline questionnaire and six preparatory and coping planning assignments; and (2) an action planning plus (AP+) program that extends the AP program by providing tailored feedback at eleven time points after the quit-attempt. The efficacy of the programs is compared to a control group (with no intervention).
Moreover, we aim to assess possible dose-response relationships between abstinence and adherence to the number of program elements. First, we expect both programs to be more effective than the control group in fostering continued abstinence twelve months after the start of the study (hypothesis 1). We expect the AP+ program to be the most effective. Moreover, we expect to find a dose-response relationship between continued abstinence and intervention-dose (hypothesis 2). Finally, we will provide an overview of the respondents' program evaluations."

3b-i) Bug fixes, Downtimes, Content Changes

4a-i) Computer / Internet literacy

4a-ii) Open vs. closed, web-based vs. face-to-face assessments:

This trial was purely web-based, respondents did not have face to face contact with the study team. Relevant sections with regard to recruitment strategies and the sort of trial (pag4): "Smokers were recruited by ads in local newspapers, 10,000 flyers distributed in the city of Maastricht, and online ads on the websites of national health funds, a national news page and the Dutch Foundation for a Smoke-free Future. The ads referred the respondents to our research website (www.sq4u.nl) for more information. All data were gathered via the Web and there was no face to face contact with the study team."

4a-iii) Information giving during recruitment

4b-i) Report if outcomes were (self-)assessed through online questionnaires

The outcome data were gathered using self-assessed online questionnaires. This is explained in the 'measures' section of the Methods. Relevant section (pag 8): Continued abstinence at the twelve-month follow-up was measured by asking whether the respondent had smoked since the quit-date.

4b-ii) Report how institutional affiliations are displayed

5-i) Mention names, credential, affiliations of the developers, sponsors, and owners

5-ii) Describe the history/development process

5-iii) Revisions and updating

5-iv) Quality assurance methods

5-v) Ensure replicability by publishing the source code, and/or providing screenshots/screen-capture video, and/or providing flowcharts of the algorithms used

5-vi) Digital preservation

5-vii) Access

Relevant sections in the Methods of the manuscript with regard to how the respondents accessed the application (pag 5 and pag 6): "Respondents registered via the research website and made their own login account (each time they were invited for participation they received this account in the invitation mail)." and "Respondents who completed all parts of their assigned SQ4U-variant (including those in the control group) were eligible to win one of twenty €250 prizes."

5-viii) Mode of delivery, features/functionalities/components of the intervention and comparator, and the theoretical framework

The section 'intervention materials' in the Methods of the manuscript (pag 6 and pag 7) provides detailed information with regard to the features and components of each of the sub parts (computer tailoring, planning components, multiple feedback moments) of the intervention. Furthermore this section explains the theoretical framework (being the I-Change Model) on which the tailored feedback was based.

5-ix) Describe use parameters

5-x) Clarify the level of human involvement

5-xi) Report any prompts/reminders used

Relevant sections in the Methods with regard to reminders (pag 5 and 6): "Respondents in the intervention groups were invited by email or SMS (optional) to perform intervention tasks (e.g., filling out a planning assignment). The same procedure was used to invite all respondents for the twelve months follow-up measurement. Reminder emails were sent when respondents did not respond to the follow-up measurement, which asked them to report on their smoking behavior using self-assessed questionnaires."

5-xii) Describe any co-interventions (incl. training/support)

There were no co-interventions in this trial.

6a-i) Online questionnaires: describe if they were validated for online use and apply CHERRIES items to describe how the questionnaires were designed/deployed

6a-ii) Describe whether and how “use” (including intensity of use/dosage) was defined/measured/monitored

6a-iii) Describe whether, how, and when qualitative feedback from participants was obtained

7a-i) Describe whether and how expected attrition was taken into account when calculating the sample size

7b) CONSORT

Not applicable

8a) CONSORT

Relevant sections with regard to random allocation of respondents (pag 4): "A software program randomly assigned a total of 2,681 respondents to one of the three conditions"

8b) CONSORT

The type of randomization was: simple randomization and is mentioned on page 4

9) CONSORT

-

10) CONSORT

Respondents were randomized by means of a software program. This is explained on pag 4.

11a-i) Specify who was blinded, and who wasn’t

-

11a-ii) Discuss e.g., whether participants knew which intervention was the “intervention of interest” and which one was the “comparator”

11b) CONSORT

Not applicable as this was an ehealth trial

12a) CONSORT

The 'analyses' section of the Methods provides detailed information with regard to all analyses conducted in this study (pag. 8 and 9)

12a-i) Imputation techniques to deal with attrition / missing values

The 'analyses' section of the Methods explains that conservative analyses in which missings at follow-up were considered as treatment failures were conducted in addition to observative analyses including complete cases only (pag 9): "For all three samples, we conducted observed case analysis (only including respondents with follow-up data) and a conservative analysis in which cases missing at follow-up were assumed to have relapsed to smoking."

12b) CONSORT

In our study we conducted modified intention to treat analyses in addition to standard intention to treat analyses (including all respondents as randomized). Furthermore, we conducted dose-response analyses. This is explained in the 'analyses' section of the Methods (pag. 8 and pag 9).

RESULTS

13a) CONSORT

The number of respondents who registered for participation, the number of those who were excluded and the number of those who dropped out was described in detail in a flow-chart.

13b) CONSORT

The number of respondents who dropped out from the study is reported in a flow-diagram. As we mentioned before we also reported on a modified intention to treat approach (in addition to the standard intention to treat approach including all respondents): in this approach respondents were excluded after randomization, this is explained in the analyses section (pag 8 and 9), the numbers are reported in the flow-chart.

13b-i) Attrition diagram

14a) CONSORT

-

14a-i) Indicate if critical “secular events” fell into the study period

14b) CONSORT

The trial was not stopped early.

15) CONSORT

The 'results' section included a table describing the baseline characteristics of the respondents and the differences in these characteristics between respondents from different conditions (pag. 10)

15-i) Report demographics associated with digital divide issues

The study reported demographics such as age, gender and education (pag. 10).

16-i) Report multiple “denominators” and provide definitions

We used different samples in this study and in all analyses the N and relevant statistical outcomes were reported.

16-ii) Primary analysis should be intent-to-treat

17a) CONSORT

95 CI is reported in all logistic regression analyses conducted .

17a-i) Presentation of process outcomes such as metrics of use and intensity of use

17b) CONSORT

The result section reported the B, odds ratios, 95% CI and p-values .

18) CONSORT

We analyzed the results using different samples. The results of all of these analyses are reported in the results section

18-i) Subgroup analysis of comparing only users

19) CONSORT

The programs did not form any harm nor unintended effects for the respondents.

19-i) Include privacy breaches, technical problems

19-ii) Include qualitative feedback from participants or observations from staff/researchers

DISCUSSION

**20-i) Typical limitations in ehealth trials**		
The Discussion contains a 'limitations' section describing the limitations of this study (pag 17 and 18): "The SQ4U-study was subject to limitations. A first limitation is that the planning assignments and feedback moments were provided at fixed times, while the varying levels of adherence found in our study suggest that programs should perhaps provide support when the respondent needs it most (i.e., by real-time support in difficult situations). Research is needed to explore the potential additional efficacy of such an approach. Second, the cut-off point for the minimum dose sample (sample III) is not based on empirical findings and needs to be explored in additional studies. Third, because of medical ethical guidelines, we could not prevent respondents from using additional help to quit smoking. The use of additional help, however, may interfere with the effects of the programs and may be beneficial or counterproductive. Further research is needed to explore which additional aids may have positive or negative effects. Finally, our study had a high lost to follow-up rate (76.9%), an issue which is very common in comparable studies [14, 23, 64, 65]. Attrition may have been caused by factors such as spam filters or invalid email accounts or because people who have quit smoking do not want to be reminded of their past smoking behavior [66]. The latter is partly supported by our data, which showed that about 60% of the respondents who dropped out of the experimental programs were non-smokers at their last visit."		
**21-i) Generalizability to other populations**		
**21-ii) Discuss if there were elements in the RCT that would be different in a routine application setting**		
**22-i) Restate study questions and summarize the answers suggested by the data, starting with primary outcomes and process outcomes (use)**		
Study questions are restated in the Discussion and a summary of the outcomes is provided (pag. 16 and 17)		
**22-ii) Highlight unanswered new questions, suggest future research**		
Other information		
**23) CONSORT**		
The Methods contain information with regard to the registration number of the trial (pag 4): "Ethical approval was obtained from the Medical Ethics Committee of Maastricht Academic Hospital and Maastricht University (MEC 08-3-003; NL21414.068.08). The study is registered with the Dutch Trial Register (NTR1892). "		
**24) CONSORT**		
The design and development protocol is published in Contemporary Clinical Trials. This is the full reference: Elfeddali, I., C. Bolman, and H. De Vries, SQ4U - a computer tailored smoking relapse prevention program incorporating planning strategies and multiple feedback moments after the quit-attempt: development and design protocol.		
**25) CONSORT**		
The section 'Acknowlegdements' contains information with regard to sources of funding (pag 19): "This study was funded by the Dutch Organization for Health Research and Innovation (grant number 6130-0030). We would like to thank Claire Jeukens and Verina Servranck for their contribution to the development and piloting of the SQ4U program materials. We would also like to thank Stivoro for their help in recruiting respondents and the respondents for their participation in the study."		
**X26-i) Comment on ethics committee approval**		
**x26-ii) Outline informed consent procedures**		
**X26-iii) Safety and security procedures**		
**X27-i) State the relation of the study team towards the system being evaluated**		
